# Editorial

**Published:** 1866-08

**Authors:** 


### Editorial.

---

Chicago Medical College.—We have received the Eighth Annual Announcement of this institution, and take pleasure in calling the attention of our readers to it. It gives a plain statement of the organization of the college; its course and plan of instruction; its clinical advantages; terms for graduation; list of students in attendance the preceding year; and the number of students and graduates in each year since its organization.

Some changes have taken place in the faculty since the last annual announcement. Prof. H. A. Johnson, one of the founders of the college, who had most ably filled, in succession, the Chairs of Physiology and Histology, and of General Pathology and Public Hygiene, has been constrained, by continued ill-health, to resign his position as an active teacher in the college. We regret this, because Prof. Johnson was one of the most thorough, eloquent, and instructive lecturers in our profession. Prof. Henry Wing has also resigned the Chair of Medical Jurisprudence. The Chair of Physiology and Histology, made vacant by the resignation of Prof. Johnson, has been filled by the appointment of Dr. John M. Woodworth, of this city, now on a visit in Europe. The Chair of Medical Jurisprudence has been filled by the appointment of Dr. R. J. Patterson, of this city, but extensively known throughout the North-west from his connection with institutions for the insane.

Dr. Patterson's attainments and previous professional career have eminently qualified him for his present position.

The next regular lecture term commences on the first Monday in October, and continues until the first Tuesday in March, following.

There are three things connected with the Chicago Medical College, to which the attention of the profession is specially directed:—First, its regular annual lecture term is five full months, with a supplementary recitation and clinical term of four months. Second, its clinical facilities and requirements; not only making hospital clinical instruction in medicine and surgery a prominent part of its daily instruction, but making attendance thereon a positive condition for graduation. In this department, the Chicago Medical College is as intimately connected with the Mercy Hospital as the Bellevue College, in New York, is with the Bellevue Hospital. Third, its increased number of professorships, with the division of the several branches into two groups, designated as junior and senior departments or courses, by which the students are arranged into junior and senior classes, and are thus enabled, each term, to confine their attention to a limited number of topics, and have their successive courses progressive instead of merely repetitional. In the practical adoption of this important principle, so long familiar and regarded as essential in all other departments of education, this college has stood alone among the medical schools of this country for the past six years. It is yet the only medical college in the country in which the student attending his first course is required to attend thoroughly to the more elementary branches of medical science, and to be examined on the same before advancing to his second or senior course. The principle lies at the foundation of all important improvements in the system of medical college instruction in our country. We are glad to see that it is, finally, attracting some attention in influential quarters. In a recent number of the *Medical Record*, of New York, the editor very properly and forcibly advocates the full adoption of this principle with a longer lecture term, by all the medical schools.

Proceedings of the Illinois State Medical Society.—We have received letters from members of the Adams Co. Medical Society, complaining that the record of the proceedings of the recent meeting of the State Medical Society, as published in the July numbers of the medical journals of this city, is incorrect, so far as relates to the communication from that Society and the action of the State Society thereon.

According to these letters, the *record* should have said that a communication was received from the Adams County Medical Society, protesting against the propriety of admitting into the State Society delegates from the "so-called Quincy Medical Society;" that the same was referred to a committee; that the committee reported a resolution declaring that the said "so-called Quincy Medical Society" was not entitled to representation, on account of its being composed in part of members who had been previously expelled from the County Society; and that this resolution was adopted. We give the proposed correction of the *record* the same publicity as the *record* itself.

In regard to the publication of the proceedings and papers read at the late meeting of the State Society and referred to the Committee of Publication, as Secretary, we feel some doubts in regard to our duty in the matter. As the representative of the Committee of Publication and Permanent-Secretary of the Society, we sent a communication to the late annual meeting, making certain recommendations in regard to the future publication of the transactions. This was referred to a committee, consisting of three prominent members of the Society, who reported adversely on our recommendations, and, after considerable discussion, both the communication and the report of the committee were laid on the table and left there, without any definite action whatever.

---

Chicago Medical Society.—Three or four of the recent weekly meetings of this Society have been occupied chiefly in discussing matters of ethics, and, consequently, have furnished less matter worthy of record than usual. At the meeting on the evening of the 29th of June, some interesting cases were reported verbally by Drs. Reid, Orrin Smith, and Wanzer, which were discussed until a late hour. At the meeting on the evening of the 6th of July, a committee reported that Dr. Jonathan W. Brooks had been proved guilty of unprofessional conduct, in relation to other members of the profession, and he was expelled from the Society.

---

European Medical News.—This is the title of a new monthly medical periodical, that Dr. Robert Stone, of New Haven, Ct., proposes to publish, as soon as a sufficient number of subscribers can be pledged to justify the undertaking. The object of Dr. Stone is, not to publish an ordinary medical journal, but a faithful abstract, monthly, of the principal medical and surgical journals of *Continental Europe.* Its size will be not less than fifty pages per month, and its price five dollars a-year. The object is a good one, and we hope it will meet with abundant success.

---

### SULPHITES IN MALARIAL FEVERS.

Moscow, Iowa, *July 18, 1866.*

Prof. N. S. Davis, M.D.:—

*Dear Sir:*—My attention being called to an article in the *American Journal of the Medical Sciences*, April No., 1866, on the remedial power of the Hypersulphite of Soda in intermittent and remittent fevers, and their analogous affections. The theory of the fermentation of the blood:—and knowing that the hypersulphite was a powerful anti-ferment, I was induced to give it a trial. Perhaps no country practitioner has used it more extensively than I, and, therefore, I only give my experience in the remedy. In the last month, I have treated over one hundred cases of simple intermittent and remittent fever with this remedy alone, and in no case has there been an exacerbation after taking the remedy a reasonable length of time. I give it in 15 gr. doses, in solution with water. I have not trusted to this remedy alone in pernicious or malignant types.

Yours truly,

W. H. BAXTER, M.D.

### PHYSICIAN'S REPORT OF ST. LUKE'S HOSPITAL, FROM OCTOBER 18TH, 1865, TO MARCH 31ST, 1866.

| | |
|---|---|
| Number of patients remaining Oct. 17th, 1865, | 13 |
| " " admitted from Oct. 18th, to March 31st, | 55 |
| Total treated, | 68 |
| Number discharged, | 54 |
| " of deaths, | 3 |
| " patients remaining April 1st, | 11 |

### SEX, NATIONALITY, AND RELIGIOUS DENOMINATION OF THOSE TREATED.

| | | | |
|---|---|---|---|
| Males, | 39 | Females, | 29 |
| Bohemia, | 1 | Norway, | 1 |
| Canada, | 8 | Nova Scotia, | 1 |
| England, | 8 | Sweden, | 1 |
| Germany, | 5 | United States, | 21 |
| Ireland, | 19 | Unknown, | 3 |
| Baptists, | 3 | Methodists, | 3 |
| Catholics, (Roman,) | 27 | None, | 9 |
| Episcopalians, | 18 | Presbyterians, | 4 |
| Lutherans, | 2 | Unknown, | 2 |

### CASES TREATED.

| | | | |
|---|---|---|---|
| Abrasions of Scalp, | 1 | Fracture, Tibia, | 1 |
| Abscess, Poplitæal, | 1 | " neck of Femur, Impacted, | 1 |
| Adhesions, Pleuritic, | 1 | " Tibia & Fibula, Compound, | 1 |
| Albuminuria, | 1 | Gastritis, Chronic, | 1 |
| Anæmia, | 1 | Hypertrophy of Heart, | 1 |
| Anthrax, | 1 | Inflammation of Cervix Uteri, | 1 |
| Bronchitis, Acute, | 1 | Lying-in Cases, | 18 |
| " Chronic, | 1 | Marasmus—Senilis, with Diarrhœa, | 1 |
| Cancer of Inguinal Glands, Scirrhus, | 1 | Periostitis of Foot, Traumatic, | 1 |
| " Pancreas, Encephaloid, | 1 | " Syphilitic, | 1 |
| Caries of Finger, | 1 | Phthisis, | 2 |
| Cerebro-Spinal Meningitis, | 1 | Pneumonia, Double, | 1 |
| Congestion, Renal, | 1 | Rheumatism, Acute, | 3 |
| Debility, | 2 | " Chronic, | 1 |
| Diarrhœa, Chronic, | 3 | Stricture, Rectal, | 1 |
| Dislocation of head of Humerus, | 1 | Ulcer, Varicose, | 3 |
| Dysmenorrhœa, | 1 | Varix, | 2 |
| Ecthyma, | 1 | Variola, transferred to pest-house, | 1 |
| Eczema, | 1 | Caries of Sternum, (eloped without treatment,) | 1 |
| Fever, Typhoid, | 3 | | |
| " Intermittent, | 2 | | |

### CAUSES OF DEATH.

| | |
|---|---|
| Cerebro-Spinal Meningitis, | 1 |
| Fever, Typhoid, | 1 |
| Marasmus—Senilis, with Diarrhœa, | 1 |
| Total, | 3 |

There have been three still-births. One, the result of the mother's falling a short time before admission. In the second case, craniotomy was performed, owing to the deformity of the mother and the unusually large size of the child. In the third case, the labor was complicated with puerperal convulsions. The child was delivered by turning. The mother made a good recovery.

JNO. E. OWENS, M.D., *Physician in charge.*

Mortality for the Month of June.—The mortality report for the month of June, as compiled by the health-officer, is given below. The statistics show an increase of 126 deaths over the corresponding period of last year. The increase appears to be principally confined to accidents, consumption, convulsions, and those patients under the age of 5 years appear to have made up the greater percentage. There is no epidemic in the city, and no cases of small-pox have appeared for many months.

CAUSES OF DEATH.

| | | | |
|---|---|---|---|
| Accidents, | 12 | Fever, Congestive, | 3 |
| Asthma, | 1 | Fever, Remittent, | 4 |
| Apoplexy, | 2 | Fever, Scarlet, | 14 |
| Abscess, | 2 | Fever, Typhoid, | 13 |
| Bronchitis, | 1 | Hemorrhage, | 1 |
| Cancer, | 3 | Hydrocephalus, | 5 |
| Childbed, | 3 | Inflammation of Brain, | 7 |
| Colds, | 1 | Inflammation of Bowels, | 7 |
| Congestion of Brain, | 6 | Inflammation of Lungs, | 8 |
| Congestion of Lungs, | 3 | Intemperance, | 2 |
| Cholera Infantum, | 4 | Lockjaw, | 2 |
| Consumption, | 14 | Marasmus, | 2 |
| Convulsions, | 26 | Measles, | 18 |
| Croup, | 9 | Meningitis, | 1 |
| Diarrhœa, | 3 | Old Age, | 12 |
| Diphtheria, | 4 | Paralysis, | 1 |
| Disease of Bowels, | 1 | Poisoning, | 1 |
| Disease of Heart, | 5 | Phthisis Pulmonalis, | 5 |
| Disease of Liver, | 2 | Stillborn, | 15 |
| Disease of Throat, | 2 | Scald, | 1 |
| Disease of Spine, | 2 | Suicide, | 1 |
| Disease of Brain, | 4 | Teething, | 11 |
| Disease of Lungs, | 3 | Tuberculosis, | 3 |
| Dropsy, | 7 | Tumor, | 1 |
| Drowned, | 11 | Whooping-Cough, | 6 |
| Dysentery, | 6 | Unknown, | 41 |
| Erysipelas, | 2 | | |
| Fever, Childbed, | 3 | Total, | 319 |

Ages of the Deceased.—Under 5 years, 165; over 5 and under 10 years, 21; over 10 and under 20, 15; over 20 and under 30, 29; over 30 and under 40, 25; over 40 and under 50, 21; over 50 and under 60, 14; over 60 and under 70, 8; over 70 and under 80, 6; unknown, 15. Total, 319.

NATIVITIES,

| | | | | | |
|---|---|---|---|---|---|
| Chicago, | 157 | Canada, | 1 | Poland, | 1 |
| Other States, | 52 | Ireland, | 43 | Unknown, | 9 |
| England, | 5 | Scotland, | 5 | | |
| Germany, | 39 | Sweden, | 4 | Total | 319 |
| Denmark, | 1 | Norway, | 8 | | |

DIVISIONS.

| | | | | | | | |
|---|---|---|---|---|---|---|---|
| North, | 94 | South, | 81 | West, | 131 | Country, | 5 |
| Unknown, | 1 | Total, | 319 | | | | |

BIOGRAPHICAL DICTIONARY.—Dr. J. M. TONER, of Washington, D.C., is engaged in the preparation of a biographical dictionary of deceased American physicians. We think him eminently qualified for the performance of the task he has undertaken, and hope the profession in all parts of the country will communicate to him freely all such facts as he needs, and such patronage as will reward him for attempting to do justice to the dead.

### PREVENTION OF CHOLERA.

At a recent meeting of the New York Academy of Medicine, the following preamble and resolutions were unanimously adopted.

Without regarding them as authoritatively settling any questions of a controversal character in relation to the etiology of cholera, we cheerfully give them a place in our columns, and ask for them a careful reading:—

### PREAMBLE AND RESOLUTIONS.

*Whereas*, The New York Academy of Medicine has endeavored to promote among its own members, and throughout the medical profession, a spirit of exact and practical inquiry into the preventive and remedial treatment of epidemic cholera; therefore, be it

*Resolved*, That this Academy hereby expresses its confidence in the utility of general and specific hygienic measures as the best means of protection against the pestilential prevalence of cholera in any locality where it makes its appearance; and that the most thorough scavenging, cleansing, and disinfection are absolutely necessary means of averting this pestilence in the cities and populous towns of our country at this present time.

*Resolved*, That in the judgement of the Academy the medical profession throughout this country should, for all practical purposes, act and advise in accordance with the hypothesis (or the fact) that the cholera diarrhœa and "rice-water discharges" of cholera patients, are capable, in connection with well-known localizing conditions, of propagating the cholera poison; and that rigidly enforced precautions should be taken in every case of cholera to permanently disinfect or destroy those ejected fluids by means of active chemical agents; also that with the same object in view, the strictest cleanliness of person and premises should be enforced upon all who have charge of the sick; also, that all privies, water-closets, and cess-pools should be kept thoroughly under the control of disinfectants.

*Resolved*, That we regard the nature and causes of cholera infection, so far as the sick or their discharges can propagate it, as being so susceptible of control that there should be no fear or hesitancy in the personal care of the sick and all that pertains to them.

*Resolved*, That immediate and thorough cleansing and disinfection of all persons, clothing, and things that have been exposed to the discharges or persons of the sick with cholera constitutes the chief end and object of any rational quarantine or external sanitary police regulation against cholera.

*Resolved*, That, for the purposes here mentioned, and external sanitary police is desirable in all great maritime and river towns, but that such sanitary regulations need not seriously embarass commercial intercourse and the interests of trade.

*Resolved*, That the main source of protection against epidemic cholera at the present time is to be found in the vigilant and effective operation of sanitary measures, municipal, domestic, and personal.

*Resolved*, That the New York Academy of Medicine cordially invites the physicians of every city and village through our country to urge the immediate adoption of adequate measures of sanitary protection against the introduction and ravages of cholera, and that to this end we pledge our brethren and the public the hearty and continued coöperation of this Academy.

---

Stevens Triennial Prize.—A Prize Fund of one thousand dollars has been established by Alexander H. Stevens, M.D., Ex-President of the College of Physicians and Surgeons, New York, for the improvement of Medical Literature, on the following plan:—

Each Prize to be awarded triennially is to consist of the interest yielded by the principal fund during the previous three years, and will amount to about two hundred dollars.

The administration of the Prize is intrusted to a commission, consisting of the President of the College of Physicians and Surgeons (*ex-officio*), the President of the Alumni Association (*ex-officio*), and the Professor of Physiology (*ex-officio*), in the same institution.

The following subjects have been selected, at the request of Dr. Stevens, for the first triennial Prize under this fund:—

1st. The best means of preventing death after surgical accidents.

2d. The history of improvements in the medical art, and the means by which they are attained.

The competing essays on either of the above subjects must be sent to the President of the College of Physicians and Surgeons, New York, on or before the first day of January, 1869. Each essay must be designated by a device or motto, and must be accompanied by a sealed envelope bearing the same device or motto, and containing the name of the author.

The envelope belonging to the successful essay will be opened and the name of the author announced at the annual commencement of the College in March, 1869.

This Prize is open for universal competition.

Edward Delafield, M.D., President of the College of Physicians and Surgeons.

Alfred C. Post, M.D., President of the Alumni Association of the College of Physicians and Surgeons.

J. C. Dalton, M.D., Professor of Physiology in the College of Physicians and Surgeons.

---

Annual Prizes of the Imperial Academy of Medicine.—These were announced on the 12th December last. The Academy prise of 1000 *francs* was adjudged to Dr. Martin, its subject being Traumatic Paralysis. Baron Portal's prize of 1000 *francs*, "On the Specific Anatomical Characters, if there be any, of Cancer," was gained by M. Cornil. There were six competitors for Madame de Civrieux's prize of 1000 *francs*; the question being, The Relations between General Paralysis and Madness." M. Magnan took the prize. Capuron's prize of a like value, "The Pulse in the Puerperal State," was not adjudged. An encouragement, however, of 600 *francs* was given to M. Hemey, one of three competitors. Baron Barbier's impossible prize of 8000 *francs*, for a cure of incurable diseases, found seven candidates. A prize of 7000 *francs* was adjudged, as the nearest approach to the programme, to M. Chassaignac, as author of "Ecrasement Linéaire." M. Amussat's prize of 2000 *francs* found four candidates, but no takers; but recompenses were given to two of them. Eight works were sent in for Godard's prize of 1000 *francs;* but the Academy gave only two recompenses.—*British Medical Journal.*

---

Obituary Record.—Died, in Boston, June 21, 1866, Reuben D. Mussey, aged eighty-six. Dr. Mussey was one of the most eminent Surgeons of New England, and was for many years Professor of Surgery in Dartmouth Medical College, in his native State. About the year 1838 he removed to Cincinnati, Ohio, and was appointed Professor in the Miami Medical College, which he held until some ten years ago. He communicated a number of very valuable papers to the *American Journal of the Medical Sciences*, among others one on fractures of the neck of the thigh-bone within the capsular ligament.

At Jaffa, whither he had gone on one of those missions of love and mercy in which he delighted, on the 5th of April, Thomas Hodgkin, aged 68. Dr. H. was a physician of great talent, was a fine scholar, an accomplished linguist, and a warm-hearted philanthropist.

